# Voices of the Past: A Case Report on the Interaction Between Childhood Trauma, Social Phobia, and Psychotic Symptoms in a Transgender Male

**DOI:** 10.7759/cureus.76006

**Published:** 2024-12-19

**Authors:** Tiago Pereira, Bárbara Ferreira, Marco Gonçalves

**Affiliations:** 1 Psychiatry, Hospital Júlio de Matos, Lisbon, PRT

**Keywords:** childhood trauma, cognitive behavioral therapies, minority stress, psychosis, social phobia

## Abstract

This case report explores the interplay between childhood trauma, social phobia, psychotic symptoms, and minority stress in a 27-year-old transgender male. L presented with psychotic symptoms, including auditory verbal hallucinations and self-referential phenomena, which were accompanied by a history of childhood sexual and emotional abuse, as well as social phobia. These challenges were further compounded by experiences of stigma, rejection, and stress related to his gender identity. We explore how affective dysregulation and maladaptive cognitive patterns, potentially shaped by past trauma, might influence psychotic symptoms, particularly in the form of auditory verbal hallucinations that are sensitive to social contexts. Addressing underlying trauma-related beliefs and anxiety can help mitigate psychotic symptoms, improve social functioning, and enhance treatment outcomes, offering an integrated pathway for recovery.

## Introduction

Extensive research underscores childhood trauma as a significant risk factor for the development and persistence of psychotic symptoms, including auditory verbal hallucinations (AVH) and persecutory delusions [1]. Emotional and sexual abuse, in particular, have been strongly associated with increased susceptibility to psychotic experiences and social phobia in adulthood. These early adversities may disrupt normal neurodevelopment, leading to lasting alterations in stress response systems, emotional regulation, and cognitive processing, which collectively increase vulnerability to psychopathology [2]. Social phobia, often co-occurring with trauma-related conditions, appears to exacerbate psychotic symptoms through mechanisms such as heightened fear of rejection, anticipatory anxiety, and avoidance behaviors. This bidirectional relationship amplifies the impact of maladaptive cognitive patterns, including hypervigilance and negative self-evaluation, on symptomatology [3]. Furthermore, minority stress, arising from stigma, discrimination, and rejection linked to marginalized identities, has been identified as a distinct contributor to the onset and exacerbation of paranoia and other psychotic features, particularly in individuals identifying as part of sexual or gender minorities [4]. This report examines the complex interplay of childhood trauma, social phobia, auditory verbal hallucinations, and minority stress in a transgender male. By exploring the mechanisms underlying these interconnected phenomena, we aim to elucidate potential pathways for therapeutic intervention, with an emphasis on trauma-informed care and the integration of cognitive-behavioral approaches to address these multidimensional challenges.

## Case presentation

L, a 27-year-old transgender male, is a single individual assigned female at birth who experienced significant gender dysphoria related to secondary female sexual characteristics. This dysphoria contributed to his emotional distress and impacted his overall well-being. L had legally changed his name to a traditionally masculine name and expressed a strong desire to pursue hormonal therapy as a gender-affirmative intervention.

He presented with a long-standing history of depressive symptoms and social phobia, which began in early adolescence following persistent bullying and frequent domestic conflict. These conflicts involved frequent arguments with his parents, whom L described as conservative and lacking awareness of mental health and psychiatric care. L also reported that his father exhibited symptoms consistent with post-traumatic stress disorder (PTSD) related to his experiences in a war context. However, according to L, his father lacked insight into these symptoms and had never sought professional help, which further contributed to the strained family dynamics and a lack of emotional support within the household.

L's personal history also includes childhood sexual abuse by a sibling.

Upon observation, L presented with a tense posture, with thought content marked by self-referential ideas best described as interpretive delusional ideas following auditory verbal hallucinations involving derogatory phrases, such as accusations of being "homophobic," "lesbian," and "sexist," which he interpreted as linked to the internalized stigma associated with his conservative parents, as well as the history of violence he endured in his youth. These phenomena had a profound emotional impact, and he experienced distress, apprehension, autonomic hyperactivation, and significant behavioral consequences, leading to increased avoidance and social isolation, further exacerbating his difficulties in daily functioning.

He described heightened anxiety preceding these phenomena, with increased intensity during social interactions and in crowded environments.

L exhibited avoidant behaviors and a pervasive fear of rejection, which appear to be central traits of his personality profile. These issues significantly impaired his social and occupational functioning over the years, resulting in ongoing challenges in maintaining relationships and engaging with daily activities.

In the initial phase of treatment, the care plan included risperidone, which was titrated up to 12 mg over a period of three months to address the psychotic symptoms. However, the response was only partial in reducing these symptoms, and the persistence of significant depressive and anxiety-related symptoms necessitated further stabilization. Given the differential diagnostic considerations between depression with psychotic features and complex post-traumatic stress disorder (C-PTSD), escitalopram was introduced and titrated up to 20 mg over two months. This intervention aimed to address the affective dysregulation and intrusive symptoms that could be attributed to both diagnoses.

This adjustment led to a marked improvement in his overall clinical picture, contributing to better emotional regulation and a notable reduction in the intensity of his psychotic and anxiety-related symptoms, further supporting the therapeutic value of targeting both depressive and trauma-related dimensions of his presentation.

## Discussion

Trauma and psychosis: mechanisms of influence

Emerging literature suggests that childhood trauma, particularly emotional and sexual abuse, exerts a lasting influence on psychotic symptomatology through multiple psychological and biological mechanisms [1]. Traumatic experiences are thought to foster dysfunctional cognitive schemas, characterized by maladaptive beliefs about self-worth and pervasive distrust of others. These beliefs are associated with affective dysregulation, including hyperarousal and avoidance behaviors, which exacerbate the emotional distress that accompanies psychotic episodes [5].

Cognitive-behavioral models propose that trauma-related beliefs and emotions serve as key mechanisms in the development of psychotic symptoms, such as AVH and persecutory delusions [1]. In L’s case, early exposure to emotional abuse and social victimization likely reinforced negative self-beliefs, creating a foundation for his auditory hallucinations and paranoid ideation. His AVH episodes are notably triggered by socially charged situations, where heightened anxiety seems to escalate intrusive thoughts into perceptual experiences of derogatory voices. This aligns with findings that suggest trauma-related intrusions can develop into psychotic symptoms when reinforced by fear and negative self-evaluation [6].

The presence of social phobia in individuals with a history of trauma may further predispose them to psychotic symptoms. In L's case, social phobia predates his psychotic symptoms and appears to have intensified his paranoid thinking, particularly through anticipatory fears of social rejection. This pathway aligns with models suggesting that early social phobia and trauma-induced fears contribute to later persecutory beliefs and self-referential paranoia. Such a mechanism underscores the profound and cumulative impact of trauma, which not only disrupts emotional regulation but also solidifies maladaptive cognitive schemas that perpetuate psychotic experiences in adulthood [2].

Anxiety and auditory verbal hallucinations (AVH)

Research indicates that AVH are frequently preceded by heightened anxiety, particularly in individuals with social phobia. The quasi-perceptual nature of these hallucinations may arise from an intensification of anxious thoughts that transform into sensory-like experiences, especially when fear of rejection or humiliation is activated in social contexts [7]. For L, whose AVH episodes are often triggered by social exposure, this suggests a direct link between social phobia and the frequency and severity of AVH, as anticipated fears become misinterpreted as external, derogatory voices. This is consistent with findings that anxiety management can alleviate AVH severity, offering a promising avenue for intervention.

In L's case, hypersensitivity to rejection appears to be a significant factor in shaping his self-referential psychotic phenomena. His AVH, often featuring derogatory accusations, such as being "homophobic" or "sexist," seems to echo internalized fears and anticipated social judgment. These phenomena are likely amplified by his history of bullying, familial conflict, and childhood trauma, which may have fostered a heightened vigilance to perceived criticism or rejection. This sensitivity aligns with patterns of self-referential ideation, where neutral or ambiguous social cues are interpreted as hostile or threatening.

Role of minority stress in symptom expression

L's gender dysphoria, characterized by an incongruence between his assigned sex at birth and his experienced gender identity and significant distress related to his secondary female sexual characteristics, adds a layer of complexity to his clinical presentation. The discomfort with his assigned gender at birth can exacerbate feelings of alienation and affect his self-perception and social interactions. Anxiety linked to gender dysphoria may interact with his psychotic symptoms, particularly in social contexts where L. anticipates rejection or judgment due to his transgender identity. Minority stress may contribute to L’s paranoia symptoms, as research indicates a specific association between these factors [4]. The compounded effects of sexual minority-related social adversity and his traumatic history may have further shaped the nature of his psychotic experiences, amplifying his vulnerability to paranoia and avoidance behaviors.

For L, the intersection of social anxiety, minority stress related to his transgender identity, and past experiences of victimization may have reinforced a maladaptive cognitive schema, predisposing him to misattribute internal fears to external sources, further perpetuating avoidance and isolation.

Implications for treatment

Given the complex interaction between trauma, social phobia, and psychotic symptoms, a comprehensive, trauma-informed approach to treatment is critical. Targeting anxiety symptoms-particularly those linked to trauma-related hyperarousal-may alleviate AVH and reduce paranoia. Research supports the efficacy of cognitive-behavioral therapy (CBT) for addressing maladaptive cognitive schemas related to trauma and social phobia [1,2]. For L, incorporating trauma-focused CBT alongside social phobia management strategies may address the underlying beliefs and anxieties that fuel his psychotic experiences.

Additionally, eye movement desensitization and reprocessing (EMDR) therapy could be a valuable component of the care plan. EMDR has shown promise in processing traumatic memories and reducing the emotional distress associated with them, which could help mitigate the impact of L's childhood trauma on his current symptomatology. By targeting the traumatic experiences that contribute to hyperarousal and self-referential paranoia, EMDR may complement other therapeutic modalities, improving emotional regulation and reducing the intensity of AVH and self-referential thoughts.

Addressing social phobia within a psychotic framework can also improve overall functional outcomes. Studies show that social phobia and paranoia often share cognitive themes of negative evaluation and anticipated harm, suggesting that early intervention for these cognitions may help mitigate psychotic symptom progression [2]. For patients like L, therapy focusing on building resilience against perceived social threats may reduce reliance on avoidance behaviors and alleviate distress from AVH and paranoia in social settings.

Furthermore, gender-affirmative interventions, including access to hormonal therapy and, if desired, surgical procedures, may play a pivotal role in reducing the psychological distress associated with gender dysphoria. These interventions can enhance L’s congruence between his physical appearance and gender identity, potentially alleviating a significant source of emotional turmoil. Integrating gender-affirmative care into L’s treatment plan could therefore serve as a complementary strategy, addressing a critical aspect of his identity and emotional well-being.

## Conclusions

L’s case underscores the intricate interplay between childhood trauma, social phobia, psychotic symptoms, minority stress, and gender dysphoria. His early experiences of abuse, familial conflict, and rejection likely contributed to the development of maladaptive cognitive schemas, such as pervasive distrust of others, hypervigilance to perceived criticism, and negative self-worth. These schemas underpinned his interpretive delusional ideas and AVH, which were particularly triggered in socially charged settings where anxiety and fears of rejection were heightened. Minority stress linked to his transgender identity and gender dysphoria further exacerbated his social isolation and self-referential thoughts. Significant improvement in L’s hallucinatory activity was observed when treatment targeted his depressive and anxiety-related symptoms. The addition of escitalopram alongside risperidone addressed the affective dysregulation and intrusive thoughts, leading to a marked reduction in the frequency and intensity of AVH.

Addressing these interconnected challenges requires a multifaceted and integrative approach. Trauma-informed interventions such as CBT and EMDR can target the core maladaptive beliefs and hyperarousal stemming from trauma, while tailored strategies for social phobia can reduce anticipatory anxiety and improve functional outcomes. Concurrently, gender-affirmative care, including hormonal therapy, can address the psychological distress associated with gender incongruence, fostering alignment between identity and self-perception. This integrative framework provides a comprehensive pathway for emotional stabilization, symptom reduction, and overall recovery.
